# Mapping optimal orthodontic implant sites in the palate using cone-beam computed tomography

**DOI:** 10.3389/froh.2024.1453665

**Published:** 2024-10-02

**Authors:** Zhengxian Zhu, Lin Zhong, Yicheng Zhao, Xiaoting Wang, Wenhao Qian, Niansong Ye

**Affiliations:** ^1^ Department of Orthodontics, Shanghai Xuhui District Dental Center, Shanghai, China; ^2^ Department of Stomatology, Shanghai Jing'an District Central Hospital, Shanghai, China; ^3^ Department of Oral Prothodontics, Stomatological Hospital of Tianjin Medical University, Tianjin, China; ^4^ Department of Implantology, Shanghai Xuhui District Dental Center, Shanghai, China; ^5^ Department of Orthodontics, Shanghai Hua Guang Private Practice, Shanghai, China

**Keywords:** palatal thickness, cortical density, orthodontic mini-implants, cone-beam CT, mini-implant anchorage

## Abstract

**Objective:**

To measure the palatal soft tissue thickness and cortical bone density to determine safe regions for the placement of orthodontic mini-implants and to examine the influence of sex and age on soft tissue thickness and cortical bone density.

**Materials and methods:**

Cone-beam computed tomography images of 42 patients (22 males and 20 females), including 21 adults and 21 adolescents, were examined in this study. The palatal soft tissue thickness and cortical bone density were measured at the coronal planes between the premolars (P4–5), between the second premolars and first molars (P5–6), and between the first molars and second molars (P6–7).

**Results:**

The thickness of the soft tissue revealed similar coronal planes, but the bone density varied. The mean thickness was 3.8 mm at 0°–60° and 1.5 mm at 60°–90°. P4–5 had the highest bone density (>600 HU), decreasing toward P6–7 (<600 HU). Bone density decreased from 90° to 0° coronally, whereas the soft tissue thickness increased. Age, sex, and their interaction affected bone and soft tissues.

**Conclusions:**

In general, areas with a high bone density tended to have thin soft tissue coronally, thus the preferred implant site tends to be more anterior to the P4–5 plane and closer to 60°–90°. Considering individual variances, mapping of the recommended regions for palatal mini-implants is suggested.

## Introduction

**Figure 1:**
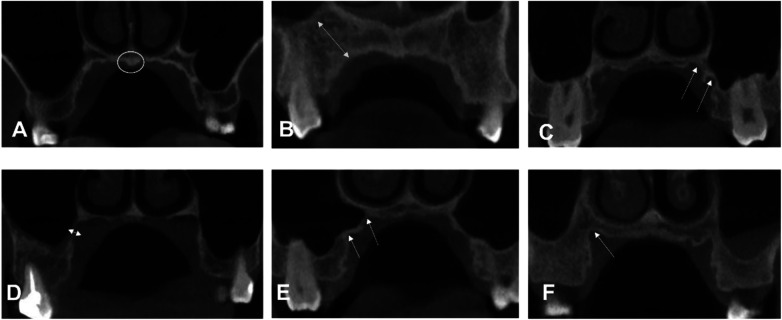
Examples of different palatal morphologic characteristics from our study sample. **(A)** Torus palatinus. **(B)** The long distance from the maxillary sinus. A manifestation of **(C,E)** two crests **(F)** one crest. **(D)** Bone region very close to the maxillary sinus.

Several orthodontic treatments require palatal mini-implants as anchorage, such as distalization of the molar (1), maxillary skeletal expansion of the arch (2), palatal closure of the extraction space (3), and intrusion of the molar (4), with minimally invasive and simple implantation performed by clinicians and less reliance on patient cooperation, as mini-implants serve as a stable source of anchorage (5). The palatal region is preferred by orthodontists over the narrower buccal interradicular space for many reasons. First, the palatal anatomy reduces the risk of root proximity to the mini-implants during insertion, which has been suggested to be the major factor (6) in mini-implant failure. Second, the palatal mini-implants secure tooth distalization, whereas those inserted in buccal bones may interfere with root movement. Third, the palatal surface is covered with a dense keratinized gingiva, which minimizes mucosal irritation and facilitates the primary stability (7, 8). A thinner mucosa improves the stability of mini-implants (9), with less likelihood of inflammation, and biomechanically decreases the moment arm of the mini-implants in the mucosal collar (10, 11). However, the palatal mucosa was three times thicker than the buccal alveolar mucosa (12) and the palatal mini-implant failure rate was as high as 10.5% (13). In addition, there was individual diversity in the palatal morphology observed in our study (Figure 1), as reported in the literature (14, 15). Other risk factors that have been reported to affect stability include poor bone quality and quantity (16), and narrow root proximity (6).

Some studies (12, 17, 18) have investigated the anatomical features of cortical and soft tissue thickness; bone quality over quantity played a dominant role in the success rate of mini-implants because increased cortical thickness did not increase pullout strength (19). Furthermore, cortical thickness appeared to plateau by the age of 16 and was not significantly associated with sex (20). However, few studies have assessed the effects of mucosal thickness and bone density on mini-implant insertions. Finally, cone-beam computed tomography (CBCT) is a non-invasive and accurate method for measuring palatal mucosal thickness (21). Therefore, this study aimed to measure mucosal thickness and bone density, identify the influence of age and sex on them, and draw safe zone maps for clinicians to plan mini-implant insertion.

## Materials and methods

### Participants

**Figure 2:**
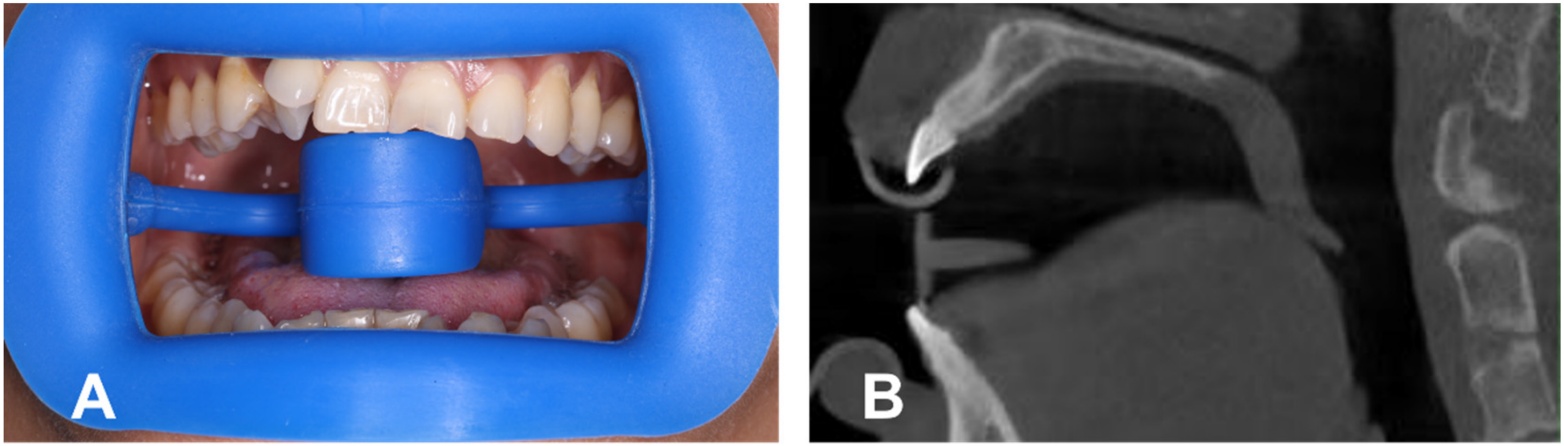
**(A)** Wearing a mouth opener, with the tongue under the tongue stop. **(B)** Screenshot of CBCT with the opener.

The study sample included subjects seeking orthodontic treatment whose signed informed consent was obtained; the protocol was reviewed and approved by the Medical Research Project of Shanghai Xuhui District Dental Center (SHXYF202205). The sample consisted of the CBCT images of 42 healthy patients. The exclusion criteria were (1) a cleft palate or lip, (2) jaw pathological lesions, (3) previous palatal treatment (palatal expansion and insertion), (4) skeletal class II and III malocclusion, and (5) palatal surface tongue contact (Figure 2).

### CBCT examinations

To obtain a clearer view of the palatal mucosa, each patient wore a plastic retractor during the filming process, with the tongue placed under the tongue holder to keep it from touching the palatal vault (Figure 2). CBCT data were obtained using a three-dimensional volume scanner (NewTom VGI, Cefal, Italy). The following settings were used: a tube voltage of 110 kV and a tube current of 1–32 mA.

### Measurements of palates

**Figure 3:**
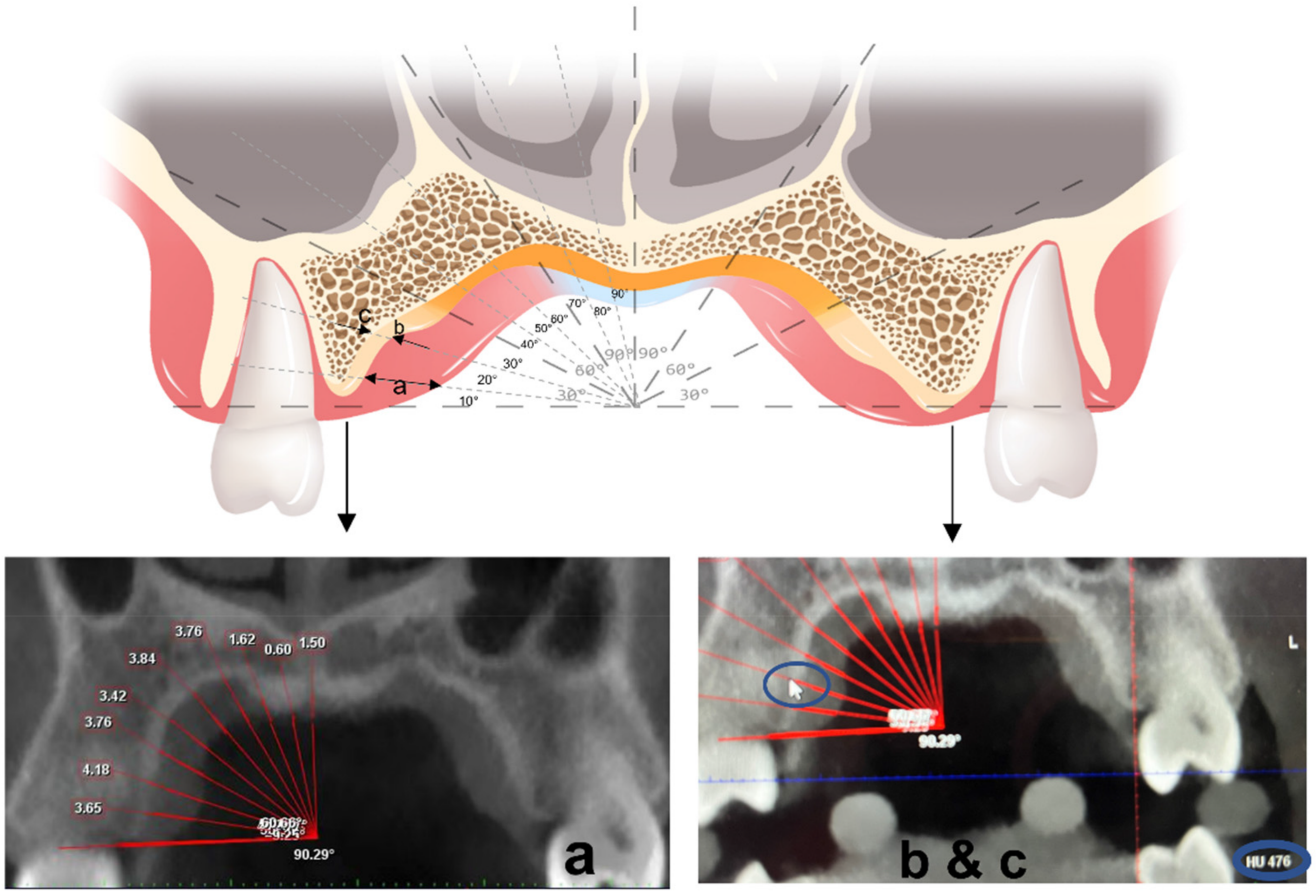
Measuring at a coronal plane: the arrow in **(a)** represents the thickness of soft tissues. The arrows in **(b,c)** represent the measured density of the medial-lateral edge of the bone cortex.

A reference line was defined as the horizontal line drawn through the alveolar ridge in the coronal plane. The palatal mucosal thickness and cortical bone density of the left and right sides were measured respectively from 10° to 90° above the reference line at 10° intervals. Every 30° were taken as the observed areas, i.e., the lower, middle, and upper sections. The mean value of each section was calculated as the average for every 30° (Figure 3).

### Statistical analyses

Intraclass correlations were used to test the reliability of the measurement method, and all measurements were repeated twice, 1 week apart. Two-way analysis of variance (ANOVA) was used to compare palatal soft tissue thickness and cortical bone density among the three planes (P4–5, P5–6, and P6–7) and among different angles (0°–90°) away from the midpalatal sutures. One-way ANOVA was applied to analyze the differences in palatal thickness of the same measurement region among the different sex and age groups. Factorial design ANOVA was used to determine interactions between age and sex. All data were analyzed using SPSS 25.0 (IBM, USA) and GraphPad 9.0 (Dotmatics, USA), and a *p*-value <0.05 was considered statistically significant.

## Results

Intraclass correlations (soft tissue thickness, *r* = 0.99; bone density, *r* = 0.92) suggested the results had high reliability. As there were no statistical differences between the left and right sides for soft tissue thickness (*p* > 0.05) and bone density (*p* > 0.05), the measurements of both sides were integrated for statistical analysis. Two-way ANOVA with repeated measures showed that bone density was significantly affected by different coronal teeth planes (P4–5, P5–6, and P6–7) (*p* < .001) and different sites away from the alveolar ridge (0°–30°, 30°–60°, and 60°–90°) (*p* < 0.001). Soft tissue thickness showed no statistical differences for different coronal planes (*p* = 0.117) but differed statistically for different sites (0°–30°, 30°–60°, and 60°–90°) (*p* < 0.001).

**Figure 4:**
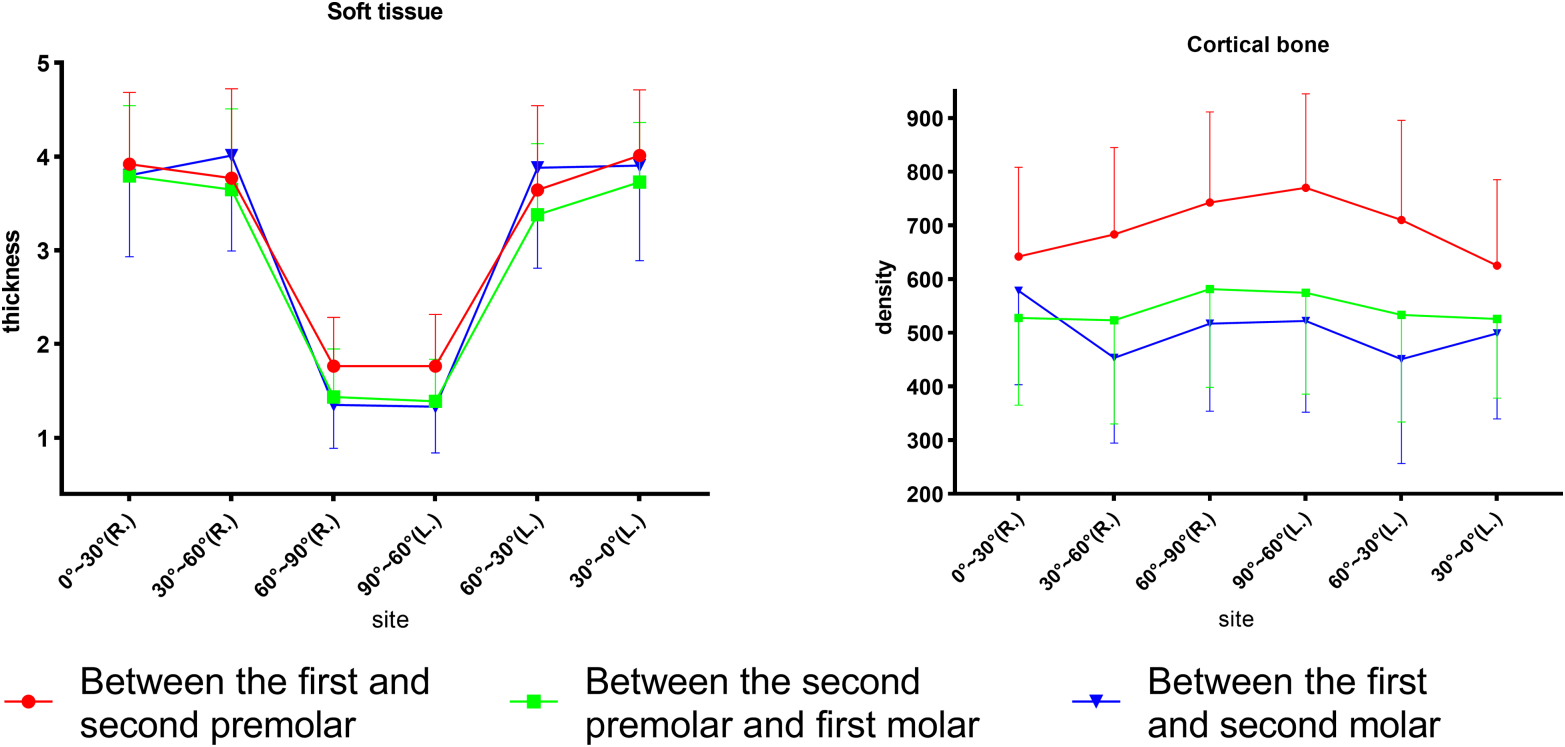
Comparison of the palatal thickness and bone density of different sites and different planes.

As displayed in Figure 4, for different sites away from the alveolar ridge, *post-hoc* one-way ANOVA showed that, at P4–5, bone density increased from site 0°–30° to site 60°–90° (*p* < 0.001), where it reached its highest density, and soft tissue thickness decreased from point 0°–30° to point 60°–90° (*p* < 0.001). At P5–6, bone density increased slightly from site 0°–30° to 60°–90° and did not reach statistical significance (*p* > 0.05), and soft tissue thickness decreased from site 0°–30° to site 60°–90° (*p* < 0.001). At P6–7, bone density ranked highest at site 0°–30°, decreased from site 0°–30° to site 30°–60°, and then slightly increased to site 60°–90° (*p* < 0.001), and soft tissue thickness decreased from site 0°–30° to site 60°–90° (*p* < 0.001). Significant differences were found from site 30°–60° to site 60°–90° for bone density among the three planes (*p* < 0.001). The three planes were similar for the soft tissue.

**Figure 5:**
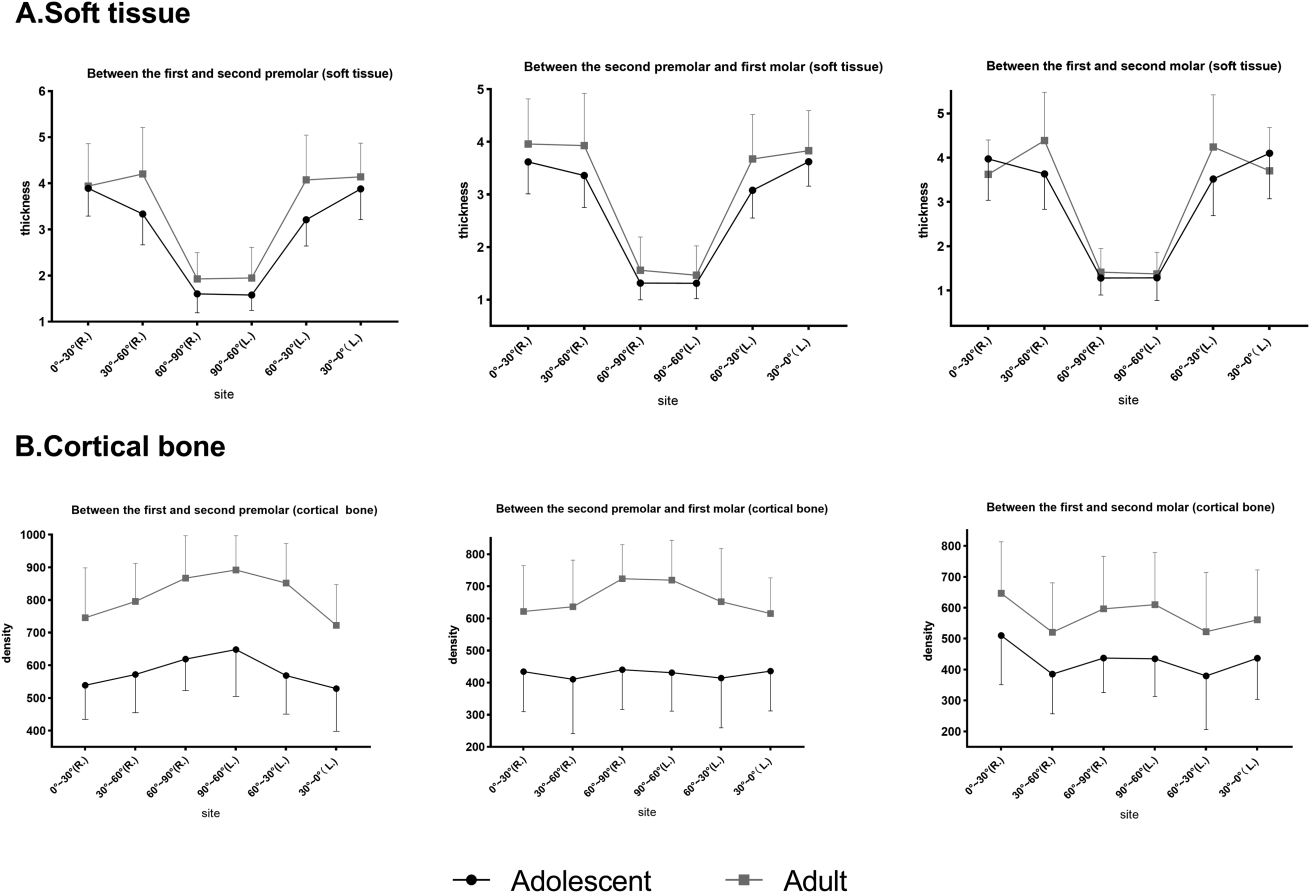
Comparison of soft tissue thickness and bone density at different planes in adults and adolescents. **(A)** Soft tissue. **(B)** Cortical bone.

Between adults and adolescents (Figure 5), two-way ANOVA revealed that the cortical bone of adults was denser than that of adolescents in all planes (P4–5 and P5–6, *p* < 0.001; P6–7, *p* = 0.001). Soft tissue thickness was significantly different between the age groups at P4–5 (*p* < 0.05) and P5–6 (*p* < 0.05), but not at P6–7 (*p* > 0.05).

**Figure 6:**
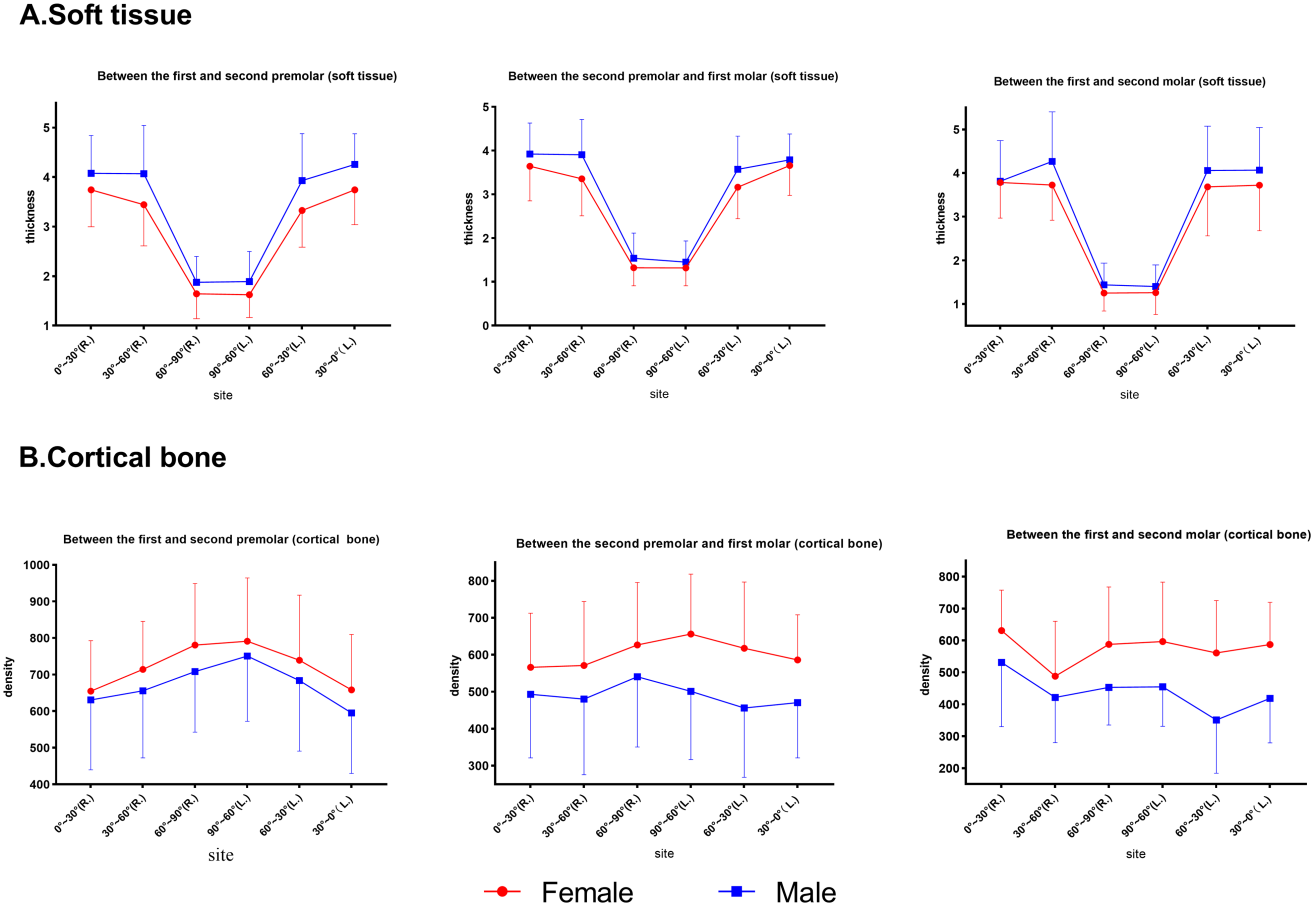
Comparison of the thickness and bone density at different planes at the same point in males and females. **(A)** Soft tissue. **(B)** Cortical bone.

For different sex groups (Figure 6), two-way ANOVA demonstrated that the soft tissue of males was thicker than that of females at P4–5 (*p* = .015), whereas P5–6 and 6–7 showed no significant difference. The bone density of females was higher than that of males at P6–7 (*p* = 0.002) and P5–6 (*p* = 0.018), but it did not reach statistical significance at P4–5 (*p* > 0.05).

**Figure 7:**
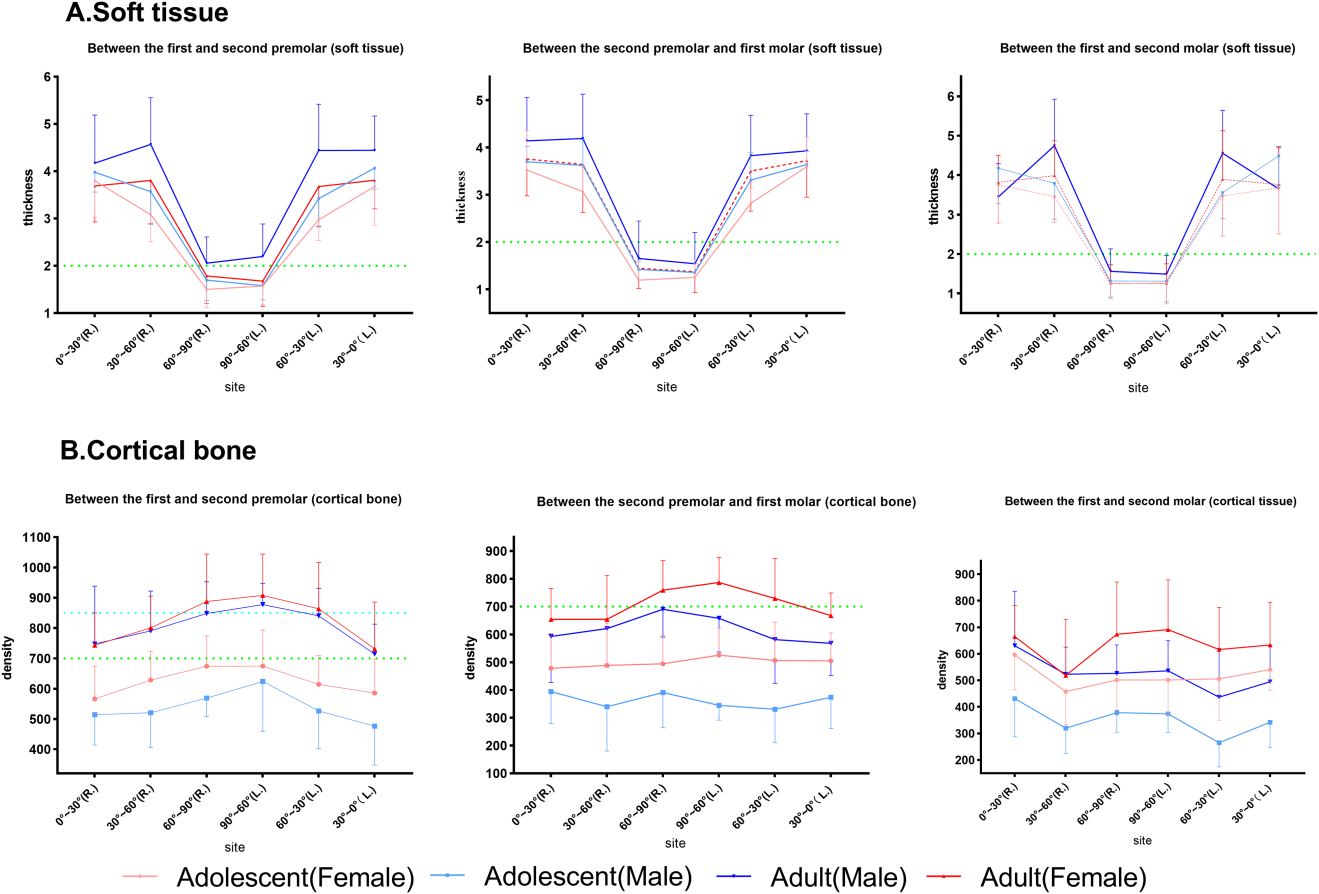
The effect of the interactions between sex and age on palatal soft tissue thickness and cortical bone. The horizontal dotted lines at the thickness of 2 mm and density of 700 and 850 HU are highlighted in each subgroup. **(A)** Soft tissue. **(B)** Cortical bone.

For the interactions between age and sex (Figure 7), factorial ANOVA showed that there were effects between age and sex on cortical bone density at P4–5 (*p* < 0.001), P5–6 (*p* < 0.001), and P6–7 (*p* < 0.001). However, no interactions were found for soft tissues, except for P4–5 (*p* = 0.002). Specifically, male adults had the thickest soft tissue and female adults had the densest cortical shell compared with the other subgroups.

In summary, the soft tissue thickness revealed similar coronal planes but the bone density varied. In addition, 0°–60° had a mean thickness of 3.8 mm, whereas 60°–90° had a mean thickness of 1.5 mm. P4–5 had the highest bone density (>600 HU), decreasing toward P6–7 (<600 HU), as shown in Tables 1 and 2. Bone density decreased from 90° to 0° coronally, whereas soft tissue thickness increased, exhibiting a statistical graph trend resembling the shape of the letter “V”. Age, sex, and their interaction affected bone and soft tissues.

**Table 1:** Descriptive statistics for palatal soft tissue thickness (mm).

Teeth plane		Mean	SD	*N*	95% CI
0°–30° R	45	3.92	0.77	42	3.68–4.16
	56	3.79	0.75	42	3.55–4.03
	67	3.80	0.87	42	3.56–4.04
	Total	3.84	0.79	126	
30°–60° R	45	3.77	0.95	42	3.48–4.06
	56	3.65	0.86	42	3.36–3.93
	67	4.01	1.02	42	3.72–4.30
	Total	3.81	0.95	126	
60°–90° R	45	1.77	0.52	42	1.61–1.92
	56	1.44	0.51	42	1.28–1.59
	67	1.35	0.46	42	1.20–1.50
	Total	1.52	0.53	126	
90°–60° L	45	1.76	0.55	42	1.61–1.92
	56	1.39	0.45	42	1.24–1.54
	67	1.33	0.50	42	1.18–1.49
	Total	1.49	0.53	126	
60°–30° L	45	3.64	0.90	42	3.36–3.92
	56	3.38	0.76	42	3.10–3.66
	67	3.88	1.07	42	3.60–4.16
	Total	3.63	0.93	126	
30°–0° L	45	4.01	0.70	42	3.77–4.26
	56	3.73	0.63	42	3.48–3.97
	67	3.90	1.01	42	3.66–4.15
	Total	3.88	0.80	126	

CI, confidence interval; SD, standard deviation.

**Table 2:** Descriptive statistics for palatal cortical bone density.

Teeth plane		Mean	SD	*N*	95% CI
0°–30° R	45	642.22	166.35	42	590.86–693.58
	56	527.97	162.72	42	476.61–579.33
	67	578.53	175.14	42	527.17–629.89
	Total	582.91	173.27	126	
30°–60° R	45	683.64	161.68	42	631.11–736.17
	56	523.62	193.45	42	471.09–576.14
	67	453.22	158.63	42	400.69–505.74
	Total	553.49	196.14	126	
60°–90° R	45	742.77	168.93	42	690.22–795.31
	56	581.69	183.25	42	529.15–634.24
	67	517.07	163.30	42	464.53–569.62
	Total	613.84	195.45	126	
90°–60° L	45	770.17	175.02	42	715.71–824.62
	56	574.93	188.97	42	520.48–629.39
	67	522.45	170.35	42	467.99–576.90
	Total	622.52	206.71	126	
60°–30° L	45	710.02	185.82	42	651.01–769.02
	56	533.17	198.79	42	474.16–592.17
	67	451.11	194.74	42	392.10–510.12
	Total	564.76	220.21	126	
30°–0° L	45	625.35	160.10	42	577.78–672.91
	56	525.69	147.55	42	478.12–573.25
	67	498.81	159.21	42	451.24–546.37
	Total	549.95	163.86	126	

CI, confidence interval; SD, standard deviation.

## Discussion

No differences were detected between the right and left sides for soft tissue and bone density (Figure 4). However, the bone density was slightly greater than that on the left at P6–7. The minor difference between the right and left molar regions could be attributed to the preferred chewing side, which had an influence on bone density differences (22). Furthermore, adult males showed a statistical difference (*p* = 0.017) between the left and right sides near the alveolar ridge section (0°–30°) at P6–7. Several studies (23–25) have reported that the density increases as the masticatory force strengthens below a certain threshold. Our sample may have a right-lateral chewing habit, and the chewing force of adult males may be stronger than that of the other subgroups. Coronal bone density decreased from the midpalatal region toward the alveolar ridge, which was similar to a previous study finding (26). However, at P6–7, the trend showed a “V” pattern in which the density decreased from the midpalatal region first and increased thereafter. That V-pattern trend could also be attributed to the increase in bone density in response to the rising chewing forces from alveolar ridges (0°–30°) in molar regions.

The mucosal thickness in the midpalate was significantly thinner than that in the other observed sections in all planes (Figure 4). From the premolar plane to the molar plane, mucosal thickness showed a decreased anatomical trend but did not reach a statistically significant difference. These results corresponded with those of previous studies showing that mucosal thickness increased laterally and anteriorly (27). The findings showed that P4–5 had the highest density, followed by P5–6 and P6-7. Choi et al. (28) similarly reported that the density increased progressively from the posterior to the anterior. This result explains the clinical recommendation of bicortical anchorage in the posterior molar region during mini-implant-assisted rapid palatal expansion (29), as the bone quality in the molar region is unfavorable for stabilizing the mini-implant.

Significant differences were observed in density and soft tissue thickness between males and females in our study. Females had a greater density and thinner soft tissue than males (Figure 6). However, a clinical study by Park et al. (30) reported that the success rate of mini-implants was independent of sex; this clinical observation contradicted our study result, which could be attributed to the younger sample included in their study. Adults had significantly greater bone density and soft tissue thickness than adolescents in our study (Figure 5), which was in agreement with a previous study report (20) that suggested a direct correlation between density and age.

**Figure 8:**
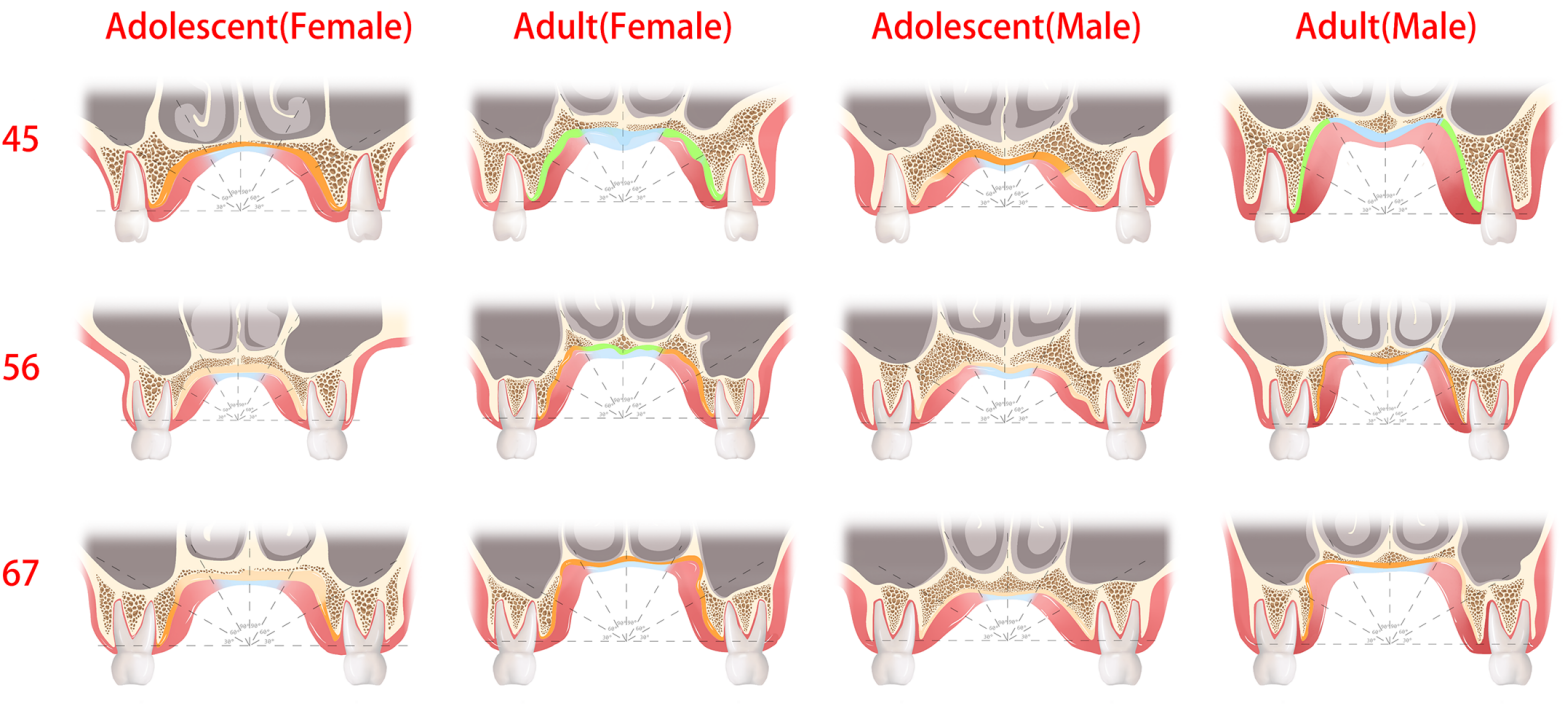
The green and blue regions are optimal sites for the placement of palatal mini-implants, where the thickness of the soft tissue was less than 2 mm or cortical bone density was the class I and II bone type. The dark yellow regions are the recommended sites for palatal mini-implants, where the bone quality was the class III bone type.

According to previous studies (31, 32), a soft tissue thickness of less than 2 mm and denser bone resulted in stronger implant stability. Norton and Gamble (33) established a quantitative bone density scale based on the Hounsfield scale, and density mean values of >850, 700–850, 500–700, and <500 HU were classified as quality I, II, III, and IV bone, respectively. In this way, between P4 and P5, the recommended locations were 60°–90°, followed by 30°–60° and finally 0°–30° (male and female adults), 60°–90° (male adolescents), and 60°–90°, followed by 0°–60° (female adolescents). Between P5 and P6, the recommended locations were 60°–90°, followed by 0°–60° (male and female adults) and 60°–90° (male and female adolescents). Between P6 and P7, the recommended location was 60°–90°, followed by 0°–60° (male and female adults), 30°–90° (female adolescents), and 60°–90° (male adolescents). Furthermore, we marked the portion of the sample with class II bone and class I bone and a soft tissue thickness of less than 2 mm as the preferred clinical reference for recommendation, as shown in Figure 7 (marked with a green line) and Figure 8. Specifically, a mucosal thickness of less than 2 mm and class II cortical portions are marked in blue, class I cortical portions are marked in green, and class III and IV cortical portions are marked in dark yellow and light yellow, respectively. Blue and green indicate the priority reference portions (Figure 8).

There are limitations in our study. First, palatal vault depth, width, and shape (Figure 1), as well as growth pattern, could be measured in future studies. Palatal bone thickness (bone depth) had great individual variation (18, 34). When the bone lacked the sufficient depth to accommodate the length and diameter of a mini-implant, bicortical anchorage was recommended by clinicians (35, 36), especially in early adolescents. In addition, the maturation of palatal sutures (37) had great variability in adolescents and young adults. Therefore, sutural ossifications must be verified using CBCT prior to implantation to avoid nasal cavity complications (38). Furthermore, the gray density values of CBCT images are not absolute (21), despite its low radiation dose, cost, superior accuracy, and spatial resolution, compared with CT. In future studies, we need to determine how to acquire the absolute value of a density threshold that enables mini-implants to obtain stability.

This study may potentially impact clinical guidelines. It is helpful for clinicians to develop more personalized implantation plans. For example, in the case of palatal bone expansion, we need to design four implantation sites. In the areas with a relatively low density, such as P5–6 and P6–7, we may need a longer or larger diameter mini-implant to enter the nasal cavity and obtain double bone cortex to increase stability. By contrast, in a high-density area, such as P4–5, we do not need a larger diameter or longer mini-implant as it will increase the torque during implantation, destroy more bone cortex, and thus decrease the initial stability. Furthermore, the anti-torsion design of the mini-implant will also undergo personalized development based on our result.

## Conclusions

• Areas with a high bone density tended to have thin soft tissue coronally. Thus, when selecting optimal implant insertion locations, bone density serves as a key consideration.
• 0°–60° showed a mean soft tissue thickness of 3.8 mm, whereas at 60°–90° the mean thickness was 1.5 mm. For 0°–60°, >8 mm-long implants are recommended.
• Adolescents had a lower bone density; therefore, it is recommended to access the nasal cavity to obtain bicortical anchorage.
• The preferred implant site tends to be more anterior to the P4–5 plane and closer to 60°–90°. Considering individual variances, mapping of the recommended regions for palatal mini-implants is suggested.

## Data Availability

The raw data supporting the conclusions of this article will be made available by the authors, without undue reservation.
